# Fungal peptidogalactomann as a biocontrol agent Against *Meloidogyne incognita* in cotton

**DOI:** 10.1590/1678-4685-GMB-2025-0162

**Published:** 2026-05-25

**Authors:** Maria Eugênia Lisei-de-Sa, José Leonardo Santos-Jiménez, Caroline de Barros Montebianco, Mariana C. Bernardino, Mateus M. Santos, Mariana Ingrid D. da S. Xisto, Andreia D. Santino, Marcelo B. Paganella, Nelson G. Oliveira, Carolina V. Morgante, Eliana Barreto-Bergter, Maria Fatima Grossi-de-Sa, Maite F. S. Vaslin

**Affiliations:** 1 Embrapa Recursos Genéticos e Biotecnologia, Brasília, DF, Brazil.; 2 Empresa de Pesquisa Agropecuária de Minas Gerais (EPAMIG), Uberaba, MG, Brazil.; 3 Instituto Nacional de Ciência e Tecnologia, INCT PlantStress Biotech, Brasília, DF, Brazil.; 4 Universidade Federal do Rio de Janeiro (UFRJ), Instituto de Microbiologia, Departamento de Virologia, Rio de Janeiro, RJ, Brazil.; 5 Incubadora de Empresas COPPE/UFRJ, Tolveg Inc., Rio de Janeiro, RJ, Brazil.; 6 Universidade Federal do Rio de Janeiro (UFRJ), Instituto de Microbiologia, Departamento de Microbiologia Geral, Rio de Janeiro, RJ, Brazil.; 7 Universidade Católica de Brasília, Programa de Pós-Graduação em Ciências Genômicas e Biotecnologia, Brasília, DF, Brazil.; 8 Universidade Católica Dom Bosco, Programa de Pós-Graduação em Biotecnologia, Campo Grande, MS, Brazil.

**Keywords:** Biocontrol, induced resistance, root-knot nematode, Gossypium hirsutum

## Abstract

Root-knot nematodes (*Meloidogyne incognita*) severely limit cotton (*Gossypium* spp.) productivity, with conventional control methods facing ecological and efficacy challenges. Here, we evaluate a fungal peptidogalactomann (pGM), derived from *Cladosporium herbarum* (commercially formulated as Hariman), as a novel biocontrol agent that primes plant defenses against nematode infection. Under greenhouse conditions, foliar application of pGM at 100 μg·mL⁻¹ resulted in an 83% reduction gall formation compared to untreated controls and yielded a four-fold increase in resistant plants (gall index = 1.5 vs. 3.2 in controls; *p* < 0.01). Nematode egg production was reduced by 60% at 120 days post-inoculation. pGM triggered a biphasic defense response, evidenced by a remarkable 40,000-fold upregulation of the systemic acquired resistance gene *PR1* and transient activation of phenylpropanoid (*PAL*) and jasmonate (*LOX2*) pathways. Notably, pGM supported healthy plant growth without phytotoxicity, contrasting with the phytotoxicity often associated with chemical nematicides. These findings indicate that pGM has strong potential as a sustainable alternative for *M. incognita* management in cotton, primarily by enhancing host immunity rather than exerting direct nematode toxicity. Further field trials are necessary to validate its effectiveness and to evaluate its integration into integrated pest management strategies.

## Introduction

Cotton (*Gossypium* spp.) is a globally significant crop, serving as the primary source of natural fiber and playing a pivotal role in the economies of many countries (FAO, 2022a). Global cotton production is projected to reach 30.6 million metric tons (Mt) in 2031, driven largely by expanded cultivation in Brazil and the United States (FAO, 2022b). However, maintaining sustainable cotton yields remains a challenge due to abiotic stresses (e.g., drought) and biotic threats, particularly plant-parasitic nematodes (Lahlali et al., 2023; Kantor et al., 2025). Among these, the root-knot nematode (RKN) *Meloidogyne incognita* (Kofoid and White, 1919) Chitwood is the most destructive, causing annual yield losses exceeding 10-20% in infested fields. Its widespread distribution and broad host range contribute to its significant impact (Xiang et al., 2017; Singh et al., 2023).

The pathogenicity of *M. incognita* begins when second-stage juveniles (J2) penetrate cotton roots, inducing the formation of giant cells through hypertrophy and hyperplasia of vascular tissues (Vilela et al., 2023;  Walia and Khan, 2023; Basavaraj et al., 2024). These galls disrupt root architecture, impairing water/nutrient uptake and predisposing plants to secondary infections by fungi (e.g., *Fusarium spp*.) and bacteria (Khan *et al*., 2023; Sikandar et al., 2025). In addition to direct damage, RKN infestation exacerbates environmental stress responses, further diminishing yield potential (Thakur et al., 2024).

Multiple strategies are employed to reduce RKN populations in the field, including crop rotation, chemical nematicides, tolerant cultivars, and biological control. However, crop rotation is limited due to the extensive host range of RKN, which includes numerous rotational field crops (Thiessen and Rivera, 2019). Synthetic nematicides, while effective, face increasing regulatory restrictions due to their toxicity to non-target organisms and environmental persistence (Abd-Elgawad, 2024). Although host resistance is an ideal approach, progress in breeding resistant cultivars has been slow, hindered by limited availability of R-genes and issues related to hybrid sterility (Abd-Elgawad, 2022; Mashavakure and Bandaru, 2023). Therefore, given the need to develop long-term and environmentally sustainable solutions, biological control agents present a promising management strategy for nematode control in agriculture (Poveda et al., 2020; Hamrouni et al., 2025).

Several microbes (bacteria, fungi, etc.) and/or their bioactive substances have been explored for RKN biocontrol (Forghani and Hajihassani, 2020; Ayaz et al., 2024; Karanastasi et al., 2024). Plant defense against pathogens is a vital trait for their survival and can be activated using inducers such as salicylic acid (SA). This compound, recognized for its role in systemic acquired resistance (SAR), has demonstrated effectiveness against various phytopathogenic species. 

Plant immune responses to nematodes involve two-tiered recognition: (1) pattern-triggered immunity (PTI), activated by conserved pathogen-associated molecular patterns (PAMPs, e.g., NAMPs for nematodes), and (2) effector-triggered immunity (ETI), mediated by host resistance (R) proteins (Kim et al., 2022; Chen et al., 2024). Preemptive induction of defenses via resistance inducers (e.g., SAR/ISR elicitors) has shown promise against RKNs (Rinaldi et al., 2021; Soares and Dias-Arieira, 2021). A study by Behzadian et al. (2025) demonstrated that the effectiveness of plant-induced resistance against root-knot nematodes (*M. incognita*) varies depending on application site and the extent of salicylic acid (SA) treatment. The results indicated that treatments targeting the roots, where the nematodes develop, resulted in a more pronounced suppression of nematode pathogenicity, emphasizing the importance of targeted application to maximize plant defense. Another promising approach for managing RKN involves the use of plant growth-promoting rhizobacteria (PGPR). Hassan et al. (2021) explored the combined inoculation of cotton or soybean seeds with *Bacillus velezensis* and pectin-rich orange peel, finding that this combination led to a 94% mortality rate in *M. incognita*. This treatment not only significantly inhibited nematode viability but also promoted plant growth, underscoring the potential of biologically active secondary metabolites in enhancing plant resistance. 

Recent advances highlight the role of fungal-derived metabolites in enhancing plant immunity. For instance, glucosylceramides (GlcCer) from *Fusarium oxysporum* reduced Tobacco mosaic virus (TMV) infection by upregulating *PR* genes (*PR-1, PR-2, PR-3, PR-5*) and enzymes involved in the phenylpropanoid pathway (*PAL, LOX, POX*) (Bernardino et al., 2020). Similar results were observed in the control of cowpea aphid-borne mosaic virus (CABMV), an aphid-transmitted potyvirus, in passion fruit using a fungal peptidogalactomann (pGM) isolated from *Cladosporium herbarum,* (Santos-Jiménez et al., 2022a , 2025). In both studies, GlcCer and Hariman positively affected plant growth by improving leaf area, plant height and root weight. 

Although most studies on Hariman have focused on viral pathosystems, its potential role in modulating host defenses against nematodes remains largely unexplored. Compared to other fungal-based inducers (Ayaz et al., 2024; Kamboj et al., 2025), Hariman presents a novel glycoprotein structure that may induce distinct signaling cascades, leading to broader defense outcomes.

Here, we evaluate the bioinput potential of this previously tested peptidogalactomann (pGM) for its ability to reduce *M. incognita* reproduction in cotton roots under greenhouse conditions. Gall index and number of eggs were reduced in Hariman-treated plants, coinciding with a strong induction of three SAR-related gene expressions. We hypothesize that Hariman activates PTI/ETI pathways, impairing nematode development while maintaining plant growth, an approach aligned with sustainable cotton production.

## Material and Methods

### Plant material and experimental design

All the experiments were performed in a greenhouse of EMBRAPA CENARGEN located at Brasilia, Distrito Federal, Brazil. Pots of 15 l were filled with a sterilized mixture of soil, sand and substrate (1:1:1) and organized on rows to test the effect of pGM (commercial formulation: Hariman) treatment on the stimulation of nematode protection in cotton. Two independent experiments were performed. During all experiments, cotton plants were maintained by following standard recommendations for watering and fertilization throughout their growth cycle.

Each experiment consisted of 30 cotton plants subjected to 100 mg.µL^-1^ of pGM treatment and 30 plants to water foliar sprays, 18 days after sown. Three days after the first application (commercial formulation: Hariman), roots of all plants were inoculated with 3,000 second-stage juveniles (J2) of *Meloidogyne incognita*. A second and a third applications of pGM were carried out 15 and 30 days after the first application, respectively. As healthy control 30 plants did not receive nematode or any spray. Nematode infection control consisted of 30 plants that received nematode inoculation but were not sprayed with water and/or pGM. To evaluate the effect of pGM in the absence of biotic stress, 10 plants were sprayed three times with pGM (Hariman formulation), and 10 plants were sprayed three times with water. These plants were not inoculated with nematode. Samples from this treatment were used for RT qPCR assays. 

To prevent cross-contamination of the peptide treatment among different groups, plants in each treatment were physically separated using rigid paper barriers. Additionally, healthy plants that did not received any spray also served as a buffer zone between pGM-treated plants. This experimental design ensures precise evaluation of the effects of peptide-based treatments and nematode inoculation on cotton plants while minimizing potential interference between treatments.

### *Meloidogyne incognita* inoculum preparation

*Meloidogyne incognita* population was multiplied on *Lycopersicon esculentum* cv. ‘Santa Clara’ and kept in a greenhouse under temperatures ranging from 23 °C to 28 °C. After three months the suspension of eggs was obtained according to the methodology of Hussey and Barker (1973) by grinding the roots in a blender with 0.5% sodium hypochlorite for approximately 30 seconds. The second stage juveniles (J2) were obtained by hatching of nematode eggs in modified Baermann funnels collected during one week under 25 °C (Flegg, 1967). The inoculum was counted using a Peter’s slide and calibrated with dilutions.

### Nematode infestation in cotton

Each plant was inoculated with 5 mL of a suspension containing 3,000 J2 of *M. incognita* by placing the nematode into three 2-cm-deep holes adjacent to the plant stem. After 90 days, the roots were carefully removed from the pots, washed and visually scored for gall incidence. The nematode reproduction was estimated by the number of eggs/g of root after 120 days after inoculation (DAI)

### Peptidogalactomann obtaining and-treatment

**Figure 1 -:**
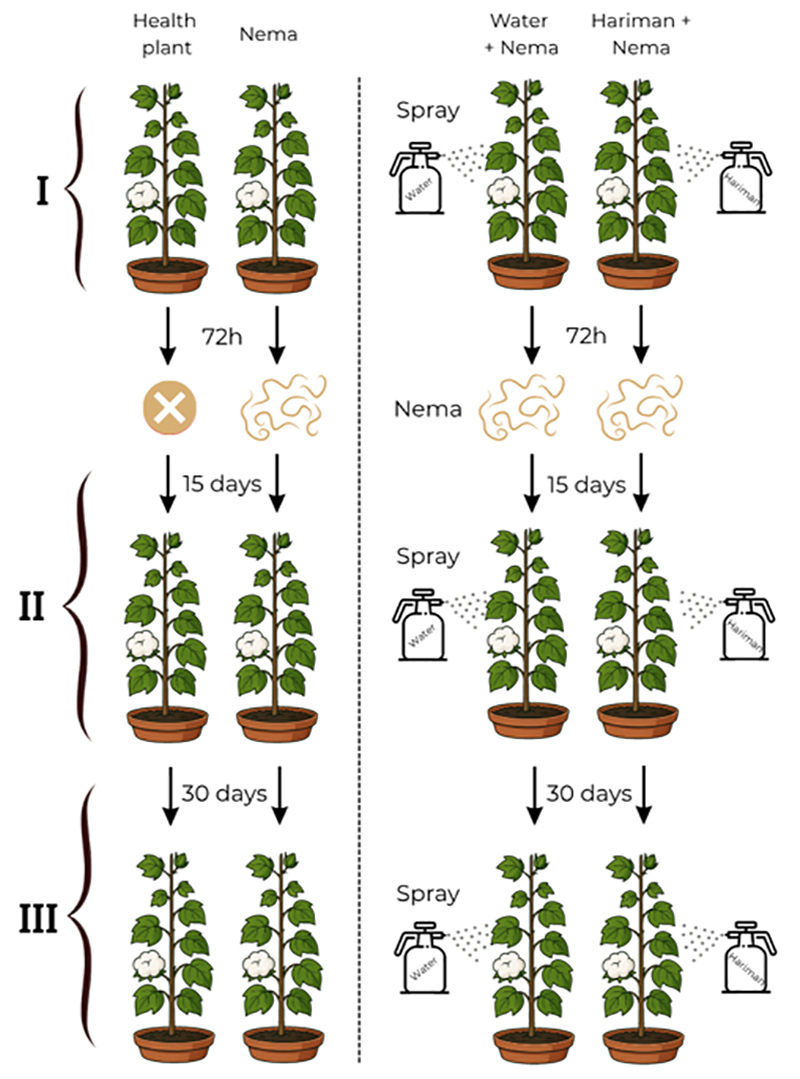
Experimental design illustrating the timing of pGM (Hariman) and water treatments in cotton plants with or without *Meloidogyne incognita* inoculation. Plants were divided into four groups: Healthy (no spray, no nematodes), Nema (nematodes only), Water + Nema (water spray and nematodes), and pGM + Nema (pGM spray, commercial formulation: Hariman, and nematodes). Treatments were applied at three timepoints: 0 (I), 15 (II), and 30 days (III). Nematode inoculation occurred 72 hours after the first spray.

A peptidogalactomann (pGM), the active compound of a commercial formulation (Hariman) was obtained from *C. herbarum* fungus strain CBS 121621 as described earlier (Mattos et al., 2018; Santos-Jiménez, 2022 a). The fungus was grown in potato dextrose broth medium (PDB) for seven days and 3MM paper filtered to obtain the fungal mass. Fungal mass was processed according to Haido et al. (1998) and Mattos *et al*. (2018). In brief, crude extracts were obtained from mycelia with hot phosphate buffer extraction (50 mM, for 2 h, at 100 °C) followed by concentration at reduce pressure and ethanol precipitation. In all experiments, cotton plants were foliar sprayed three times with pGM at 100 μg·mL⁻¹, using the commercial formulation (Hariman) resuspended in tap water, when plants were at the early vegetative stage (three to four true leaves). An interval space of 15 days was adopted between each spray. The dose concentration was based on Santos-Jiménez *et al*. (2022a). The treatments were performed by pulverization of Hariman performed with a high-pressure device W550, Wagner. As a control, tap water was foliar sprayed using the same procedure (water treatment). Part of the pGM- or water treated plants were inoculated with *M. incognita* and part didn’t receive any nematodes, as shown in experiment design shown in Figure 1.

### Evaluation of pGM treatments over *M. incognita* reproduction

In the first experiment, conducted at 90 days after inoculation (DAI), cotton plants were evaluated using a modified version of the Taylor and Sasser (1978) scale, based on the gall index (GI), which ranges from 1 to 5: (1) resistant - fewer than 40 galls per root system; (2) moderately resistant - 41 to 80 galls per root system; (3) moderately susceptible - 81 to 120 galls per root system; (4) susceptible - 121 to 160 galls per root system; and (5) highly susceptible - more than 160 galls per root system.

In the second experiment, at 120 DAI, plants were assessed based on the total number of eggs, following the methodology of Coolen and D’Herde (1972). For egg extraction, cotton roots were blended in a 0.5% sodium hypochlorite solution. The number of eggs and second-stage juveniles was quantified using a Peters’ counting slide under a light microscope.

### Evaluation of pGM treatment on cotton growing in greenhouse conditions

To observe if pGM treatment of cotton could impact plant grow and development in green house conditions, the plant height, the number of leaves and number of flower buds of the nematode inoculated treated and controlled (healthy, only nematode inoculated and water treated + nematode inoculated) plants were evaluated at 30, 60 and 90 days after the first spray. Height was measured with the aid of a measuring tape and the leaves and flowers buds were manually counted.

### Analysis of key gene expression by RT-PCR

Leaf samples for gene expression analysis via quantitative polymerase chain reaction (qPCR) were collected three days following each spray application. For this purpose, three leaves were harvested from three individual plants. Subsequently, these samples were submerged in liquid nitrogen and stored at -80 °C to preserve RNA integrity. 

The expression of three defense-related genes was evaluated: PR1 (Pathogenesis-Related protein 1), PAL (Phenylalanine Ammonia-Lyase), and LOX2 (Lipoxygenase 2). Total RNA was extracted using Direct-zol RNA MiniPrep (Zymo Research, Irvine, CA, USA) following the manufacturer’s instructions. cDNA synthesis was performed with SuperScript™ VILO™ Master Mix (Invitrogen, Waltham, MA, USA), and qPCR reactions were conducted using PowerUp™ SYBR™ Green Master Mix (Thermo Fisher Scientific, Waltham, MA, USA). Two housekeeping genes, PP2A (Protein Phosphatase 2A) and Actin, were used as internal controls for normalization. Primers are shown at Table S1.

qPCR reactions were carried out in technical triplicates using the StepOnePlus™ Real-Time PCR System (Applied Biosystems). The thermal cycling conditions were: initial denaturation at 95 °C for 2 minutes, followed by 40 cycles of 95 °C for 15 seconds, annealing at 50-61 °C (depending on the primer set, Table S1) for 1 minute, and extension at 60 °C for 1 minute. 

Cycle threshold (Ct) values were analysed using the 2^−ΔΔCt method (Livak and Schmittgen, 2001) and expressed as relative gene expression levels. Fluorescence threshold values were automatically calculated using StepOne™ Software.

### Statistical analysis

Data from cotton development parameters and nematode reproduction were analyzed using the Kruskal-Wallis test to assess statistically significant differences among treatments. When significant differences were detected, Dunn’s multiple comparison test was performed as a post hoc analysis to identify specific group differences. For gene expression analysis, a two-way ANOVA followed by the Bonferroni post hoc test was used to evaluate treatment effects. All statistical analyses were conducted using GraphPad Prism version 5.00. Results are presented as means **±** standard deviation (SD), and differences were considered statistically significant at *p < 0.05*.

## Results 

### Nematode suppression efficacy of pGM treatment

The nematicidal potential of pGM was evaluated in cotton plants (cultivar BRS 372) inoculated with *M. incognita* under greenhouse conditions. Plants aged 18-20 days, reaching the three- to four-leaf stage, were sprayed with 100 mg mL⁻¹ of pGM (commercial formulation: Hariman) or water as a control, followed by inoculation with 3,000 second-stage juveniles (J2) of *M. incognita* 72 hours later. Additional sprays were applied at 15 and 30 days after the first treatment. Two independent experiments were conducted, comprising 60 and 120 plants, respectively (Figure 1). 

**Figure 2 -:**
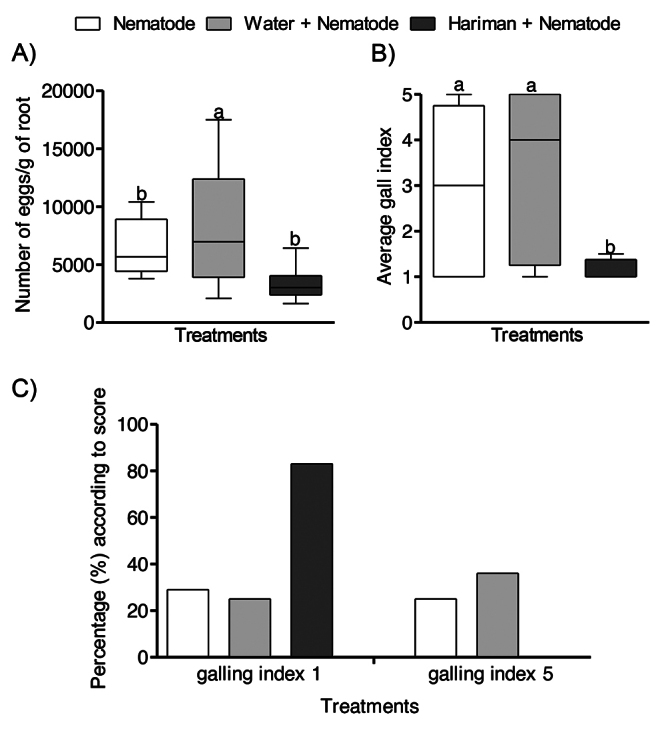
pGM (Hariman) reduces *Meloidogyne incognita* reproduction and gall formation in cotton roots. (A) Mean number of nematode eggs per gram of root at 120 days after inoculation (DAI). (B) Galling index (Taylor & Sasser scale) at 90 DAI. (C) Distribution of galling severity among treatment groups. Bars represent mean ± SD. Statistical significance was assessed using the Kruskal-Wallis test followed by Dunn’s post hoc test. Different letters indicate significant differences (*p < 0.05; p < 0.01*).

**Figure 3 -:**
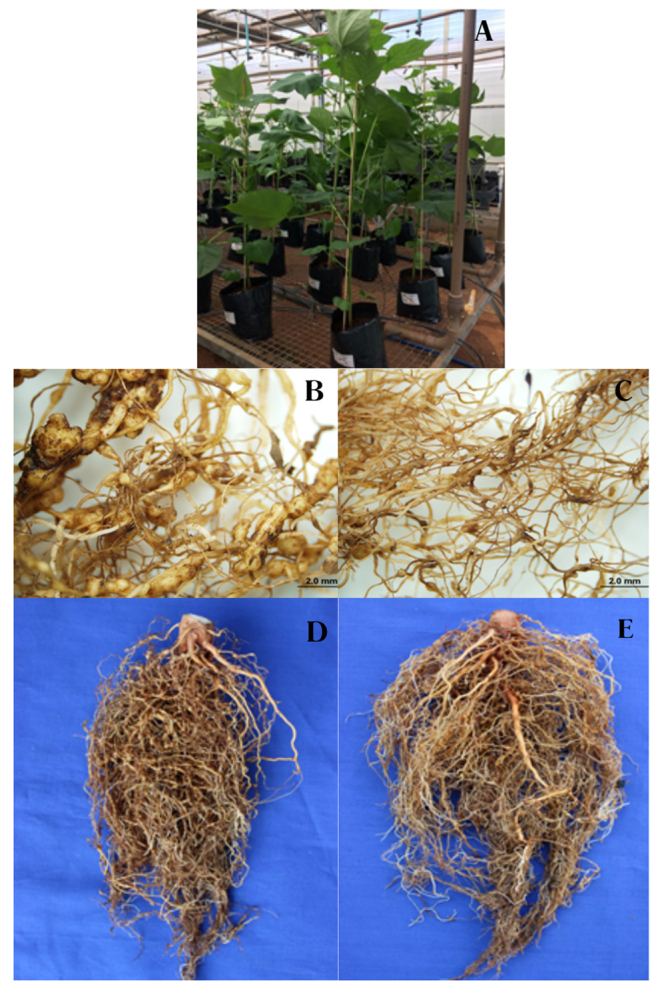
pGM (Hariman) treatment improves root architecture and reduces galling symptoms in cotton plants infected with *Meloidogyne incognita*. (A) Overview of the greenhouse experimental setup during the vegetative phase. (B) Severely galled root system from a water-treated plant inoculated with *M. incognita*. (C) Root system from a pGM-treated plant inoculated with *M. incognita*, showing minimal galling. (D) Representative root system of a water-treated, nematode-infected plant showing extensive deformation. (E) Root system of a pGM-treated, nematode-infected plant displaying preserved structure and healthy development.

pGM demonstrated high efficacy in controlling *M. incognita* infection in cotton plants. Nematode reproduction rates were reduced by 60% in pGM-treated plants at 120 days post-inoculation (*p < 0.076*) (Figure 2A ). The mean galling index for pGM-treated plants was 1.5, significantly lower than the 3.2 observed in water-treated controls (*p < 0.001*) (Figure 2B ). Additionally, gall formation assessment showed that 83% of pGM-treated plants exhibited complete resistance (galling index = 1), representing a four-fold increase compared to untreated controls, where only 20% of plants showed resistance. Notably, none of the pGM-treated plants reached the highest severity level on the galling scale (galling index = 5), while both the nematode-only and water-treated groups included plants with severe gall symptoms-highlighting their increased susceptibility to nematode infection (Figure 2C ). Visual assessment confirmed that pGM-treated roots displayed healthier architecture with minimal gall formation, contrasting sharply with the heavily galled root systems of untreated plants (Figure 3). Plants from pGM-treated *M. incognita* inoculated plants exhibited healthier roots showing clearly less galls compared to water-treated plants. Root systems of pGM-treated plants maintained normal branching patterns, with no signs of stress-induced stunting or deformation (Figure 3).

### Impact on plant growth and phenological parameters

**Figure 4 -:**
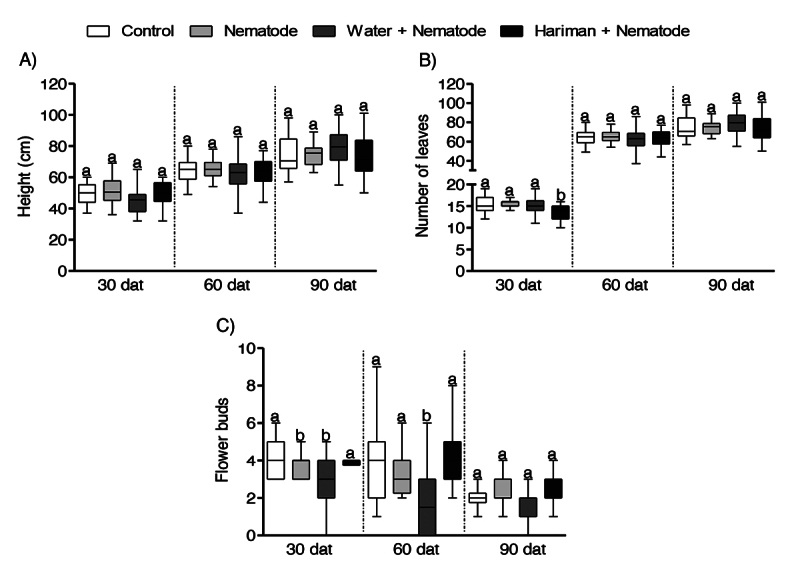
pGM (Hariman) application maintains cotton plant growth under greenhouse conditions. Morphological parameters of cotton plants at 30, 60, and 90 days after the first treatment (dat): (A) plant height (cm), (B) number of leaves, and (C) number of flower buds. Data is presented as means ± standard deviation (SD). No statistically significant differences were observed among treatments.

pGM-treated plants not only effectively controlled nematode infection but also maintained normal plant growth without any observable phytotoxic effects (Figure 4). Measurements of plant height, leaf number, and flower bud development showed no significant differences among treatments. The reduced number of flower buds observed at the final assessment (90 DAI) was likely due to suboptimal greenhouse conditions. Environmental stresses, such as temperature fluctuations and the limitations of pot cultivation, can negatively impact plant development. Although cotton is typically cultivated as an annual crop in the field, it is actually a perennial species with an indeterminate growth habit.

### pGM-treated plants showed induction of defense-related genes expression

**Figure 5 -:**
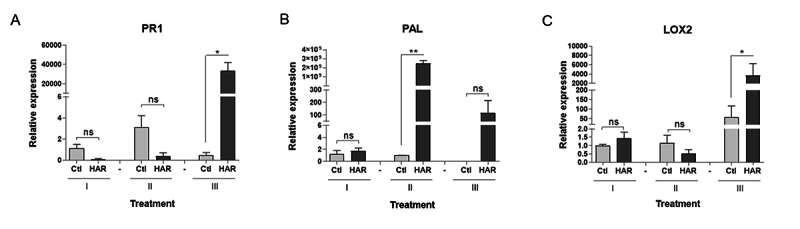
pGM (Hariman) induces expression of defense-related genes in cotton leaves. Relative transcript levels of (A) *PR1*, (B) *PAL*, and (C) *LOX2* genes in pGM-treated (HAR) and control (Ctl) plants at three timepoints: I (day 0, first spray), II (day 15, second spray), and III (day 30, third spray). Gene expression was quantified by RT-qPCR and normalized to actin and PP2A1 genes. Data are presented as mean ± SD of three biological replicates. Asterisks indicate statistically significant differences between treatments (*p < 0.05; p < 0.01*); ns = not significant.

To assess whether pGM modulates defense genes in treated cotton plants, the expression levels of two systemic acquired resistance marker genes were analyzed using RT-qPCR throughout the treatment period. Gene expression analysis provided valuable insights into the mode of action of pGM. The pathogenesis-related *PR1* gene, a marker for salicylic acid-mediated resistance, showed a 40,000-fold upregulation following the second spray (*p < 0.001*), confirming strong systemic acquired resistance activation (Figure 5). *Phenylalanine ammonia-lyase* (*PAL*), involved in phenolic compound biosynthesis, exhibited rapid but transient induction, peaking after the first spray before returning to baseline levels. *Lipoxygenase* (*LOX2*), associated with jasmonate signaling, mirrored *PR1* activation, with significant upregulation only after the second spray. These findings suggest a two-phase defense mechanism: an early phenylpropanoid response followed by robust, sustained resistance mediated by SA and JA pathways. 

## Discussion

Biological inputs (bioinputs), particularly microbial biocontrol agents, are increasingly recognized as sustainable alternatives to chemical nematicides for managing plant-parasitic nematodes in cotton. Advances in microbial ecology, plant immunity, and formulation technologies have enabled the development of highly targeted bioinputs that not only suppress nematode populations but also promote plant health and resilience (Poveda et al., 2020; Silva et al., 2024; Valente et al., 2025). Plant-growing promoting microorganisms (PGPM) as rhizobacteria specially *Azospirillum*, *Bacillus*, *Pseudomonas* and *Rhizobium* as well mycorrhizal fungi and beneficial *Trichoderma* spp. can modulate expression of stress-related genes and antioxidant enzymes, enhancing osmotic adjustment, ROS scavenging, maintenance of photosynthetic activity under stress and improving nutrient uptake (Backer et al., 2018; Enebe and Babalola, 2018; Gómez-Godínez et al., 2023). At the same time, many strains act as biocontrol agents against fungi, bacteria, and nematodes, reducing disease incidence while supporting growth and yield (El-Saadony et al., 2022; Kumar et al, 2022). The fungal peptidogalactomann (pGM) can modulate the expression of defense-related genes and phytohormones, as shown before, even being a single complex fungal molecule (Santos-Jiménez et al., 2022a , b). 

In this context, our evaluation of pGM highlights its potential use as a biotechnological tool. Unlike conventional biocontrol and/or inoculants agents which rely on rhizosphere colonization and direct parasitism, the use of biomolecules such as pGM may offers some advantages. While microbial biological control agents (BCAs) face challenges related to viability and environmental adaptability, this glycoprotein-based formulation, commercially known as Hariman, demonstrated stability under greenhouse conditions, and showed compatibility with existing agrochemicals as NPK and/or Bacillus and humus formulations (Santos-Jiménez et al., 2022b ). Besides the induction of direct antagonism through parasitism or antibiosis by (microbial biocontrol agents like Trichoderma and Bacillus (Yao et al., 2023), it was shown that they could some VOCs produced by them can induces plant defense mechanism (reviewed by Manawasinghe et al., 2025). pGM treatment activates the plant’s own defense mechanisms via immune priming as shown by qRT-PCR experiments with cotton treated plants (Figure 5) and before by Mattos et al. (2018), Santos-Jiménez *et al*. (2022a, b; 2025), were a clear induction of defense related genes and/or phytohormones was observed after treatment. The use of formulation instead of living microorganisms may confers key advantages in terms of application method, consistency, and formulation stability.

The efficacy of pGM in reducing *M. incognita* reproduction by 60% is comparable to that reported for other biocontrol strategies, yet the underlying mechanisms offer important distinctions. For example, microbial agents like *Trichoderma longibrachiatum* suppress nematode populations by producing extracellular enzymes such as serine proteases, achieving approximately 38% reduction in infectivity (Wang et al., 2024). Similarly, in commercial formulations like Micosplag^®^ (composed of dormant spores of *Paecilomyces lilacinus*, *Metarhizium anisopliae* and *Beauveria bassiana*) and Tricho-D^®^ (composed of dormant spores of *Trichoderma harzianum*) low levels of nematode infection (6-12% infection) were observed in greenhouse experiments with coffee (Castro-Toro and Rivillas-Osorio, 2022). However, both require effective root colonization and are highly sensitive to soil composition and microbiome interactions, often resulting in variable field performance. By contrast, pGM is applied direct at the leaves and does not depend on rhizosphere colonization, enabling more consistent delivery and broader environmental adaptability.

Other molecular elicitors, such as selenium nanoparticles, have shown comparable reductions in *Meloidogyne* spp. infection levels (around 60%) but are limited by narrower operational windows in terms of pH and temperature stability (Nikam et al., 2023). Bacterial biocontrol agents like *Bacillus firmus* can achieve even higher suppression rates-up to 75%-but their efficacy relies on root exudate-mediated chemotaxis and colonization efficiency, both of which are strongly influenced by soil conditions (Huang et al., 2021; Gattoni et al., 2023). pGM circumvents these limitations by acting through a host-mediated immune response, independent of microbial competition or soil variability.

Mechanistically, pGM induces a robust, biphasic activation of the plant’s immune system, characterized by an early upregulation of the marker gene SA signaling pathway PAL, followed by delayed expression of SAR-related genes such as *PR1* and *LOX2* (Wang et al., 2020; Agho et al., 2023; Soltani et al., 2023). Notably, 83% of treated plants exhibited high nematode resistance (galling index = 1.5 vs. 3.0 in controls), which correlates with a 40,000-fold upregulation of the SAR marker *PR1* after the second spray. This response mirrors defense patterns observed in cotton foliar endophytes (Zhou et al., 2020), but with greater predictability and uniformity than microbial treatments. This controlled activation likely helps minimize potential trade-offs between defense responses and plant growth. Unlike chemical SAR inducers such as acibenzolar-S-methyl, pGM-treated plants maintain growth rates and biomass accumulation comparable to those observed with biostimulants like *Trichoderma* spp. (Silva et al., 2022). Achieving this balance between defense activation and growth sustainability is essential for successful field application. This staggered defense priming mirrors patterns observed in cotton plants treated with elicitors, where phased immune activation improved long-term pathogen resistance without affecting plant development (Qiu et al., 2024; Khan et al., 2025).

The durability of the protective effect induced by pGM is another notable advantage. The delayed SAR response, marked by PR1 expression, is maintained over time, correlating with long-term nematode suppression even at 120 days post-application. This contrasts with the relatively short-lived efficacy of certain microbial metabolites, such as those produced by *Bacillus* spp., which degrade rapidly in the soil environment (Gattoni et al., 2023). Furthermore, the biochemical composition of pGM as a glycoprotein presents multiple practical benefits. Unlike microbial inoculants, it does not require cold-chain logistics (Silva et al., 2024), simplifying storage and distribution. Its foliar application bypasses competition with native soil microbiota (Poveda et al., 2020), enhancing compatibility across cropping systems. Moreover, its formulation allows for precise dosing and reproducibility, overcoming variability issues often encountered in microbial fermentation-based bioinputs (Vassileva et al., 2021; Ain, 2024; Rocha et al., 2024).

In summary, our greenhouse pGM evaluation indicated that its use may addresses several limitations of current bioinput technologies, offering a promising solution for integrated nematode management in cotton. Nonetheless, further research is needed to assess its performance under field conditions and across a broader range of nematode species, including *M. javanica*, which may exhibit different susceptibilities to plant elicitors (Castro-Toro and Rivillas-Osorio, 2022). Future studies should also explore synergistic combinations with microbial biocontrol agents, such as Trichoderma-based seed treatments, to enhance early-season protection and expand efficacy across different contexts. Thus, pGM represents a valuable addition to the growing toolbox of sustainable agricultural inputs, bridging the gap between biological effectiveness and practical deployment in diverse agroecosystems.

## Data Availability

The entire dataset supporting the results of this study has been published in the article and the supplementary materials section.

## Supplementary material

The following online material is available for this article:

Table S1 - Primers used.
