# Supplementary material for: Fungal peptidogalactomann as a biocontrol agent Against Meloidogyne incognita in cotton
Source: Genet Mol Biol. 2026 May 25;49(Suppl 3):e20250162. doi: 10.1590/1678-4685-GMB-2025-0162 (PMC13203359; doi:10.1590/1678-4685-GMB-2025-0162)
Supplement: Table S1 - [file 1415-4757-GMB-49-s3-e20250162-s1.pdf]

Genetics and Molecular Biology
https://doi.org/10.1590/1678-4685-GMB-2025-0162

Supplementary Material to "Fungal peptidogalactomann as a biocontrol agent against *Meloidogyne incognita* in cotton"

Table S1 - Primers used.

	Sequence 5'-3'	Tm	Amplicon
GhPR1_F	ACCTCAACGCTCACAACACA	61º	134
GhPR1_R	GGTCCACTGGAGTGCACAAG	61º	
GhPAL_F	CGAGGAACAAAGCATTACAT	61º	154
GhPAL_R	GTGGGAGACCGTTGTTGTAG	61º	
GhLOX2 F	AAGGCCATTTCTGACAGTG	60º	206
GhLOX2 R	AGCGAATTCCTCATCCCTA	60º	
GhActin_F	TCCCATTGAGCATGGGATCG	55º	200
GhActin_R	CGTGAGAAGAACAGGGTGC	55º	
GhPP2A1 F	GATCCTGTGGAGGATGGA	60º	200
GhPP2A1 R	GCGAAACAGTTCGACGAGAT	60º	
